# Phase I dose-escalating study of docetaxel in combination with 5-day continuous infusion of 5-fluorouracil in patients with advanced gastric cancer

**DOI:** 10.1186/1471-2407-5-87

**Published:** 2005-07-22

**Authors:** Se Hoon Park, Soo-Mee Bang, Eun Kyung Cho, Dong Bok Shin, Jae Hoon Lee, Woon KI Lee, Min Chung

**Affiliations:** 1 Division of Hematology and Oncology, Department of Internal Medicine, Gachon Medical School Gil Medical Center, Incheon 405-760, Korea; 2 Department of General Surgery, Gachon Medical School Gil Medical Center, Incheon 405-760, Korea

## Abstract

**Background:**

Published data suggests that docetaxel combined with 5-fluorouracil (5-FU) may have synergistic activity in treating advanced gastric cancer. We performed a phase I study of docetaxel and 5-FU to determine the maximum tolerated dose (MTD), the recommended dose for phase II studies, and the safety of this combination.

**Methods:**

Eligible patients had recurrent and/or metastatic advanced gastric cancer with normal cardiac, renal and hepatic function. Traditional phase I methodology was employed in assessing dose-limiting toxicity (DLT) and MTD. On day 1 every 3 weeks, docetaxel 75 mg/m^2 ^(fixed dose) was infused over 1-h, followed immediately by 5-FU as a 5-day continuous infusion.

**Results:**

Dose escalation schema was as follows: dose level (DL) 1 (5-FU 250 mg/m^2^/day), 2 (500), 3 (750), and 4 (1000). Three patients were enrolled on DL1, without DLT. On DL2, 1 DLT (grade 3 stomatitis) was developed in first 3 patients, and this cohort was expanded to 6 patients. Three patients had been enrolled on DL3. Because two out of 3 patients had DLTs, the MTD was reached at DL3.

**Conclusion:**

The recommended phase II dose of this combination is 75 mg/m^2 ^docetaxel on day 1 immediately followed by a 5-day continuous infusion of 5-FU 500 mg/m^2^/day.

## Background

Gastric cancer remains the most common cause of cancer-related death in Korea [1]. For patients with unresectable, locally advanced, or metastatic disease, chemotherapy can provide significant palliation of symptoms [2,3]. When used as single agents, 5-fluorouracil (5-FU), doxorubicin, cisplatin, and mitomycin C are considered active in gastric cancer, producing response rates in up to 20% of patients [4]. Of these, 5-FU is an central cytostatic antimetabolite with a broad range of antitumor activity in breast, gastrointestinal, head and neck, and ovarian cancers. When given as a prolonged continuous infusion, stomatitis and diarrhea are the principal toxicities, whereas myelosuppression is more commonly observed with intravenous bolus injections [5,6]. Because 5-FU has demonstrated synergistic interaction with many antineoplastic agents, it is currently most often administered in the setting of combination chemotherapy regimens. However, most trials with different combinations of these drugs provided similar survivals ranging from 6 to 10 months [7,8]. No regimen has been universally recognized as standard.

Among the new agents, docetaxel is a novel semisynthetic taxane with significant antitumor activity and manageable toxicity consisting primarily of myelosuppression [9]. Results of docetaxel-containing regimen in the treatment of gastric cancer are encouraging. Phase I and II clinical trials have confirmed that docetaxel is effective in patients with advanced gastric cancer when used as monotherapy, yielding response rates of 20% [10,11]. Docetaxel and 5-FU are highly synergistic [12], and clinical evidence suggests lack of cross-resistance.

When this study was initiated, most previous studies used docetaxel 60–85 mg/m^2 ^on day 1, followed by a 5-day continuous infusion of 5-FU 500–1000 mg/m^2^/day, every 3 weeks [12-15]. However, we had experienced severe toxicities such as febrile neutropenia and grade 3 or 4 stomatitis in treating patients with advanced gastric cancer as a practice with docetaxel 75 mg/m^2 ^and 5-FU 1000 mg/m^2^/day for 5 days. Therefore, we performed a phase I study of docetaxel and 5-FU to determine the maximum tolerated dose (MTD) and the safety of this combination.

## Methods

### Patients

This was a phase I dose-escalating study conducted between Mar 2002 and Aug 2002. The study was conducted according to the principles stated in the latest version of the Declaration of Helsinki. This study protocol was reviewed and approved by the Gil Medical Center institutional review board, and signed informed consent was obtained from all patients prior to their enrollment.

Patients enrolled into this study had to be at least 18 years of age and have a histologically confirmed diagnosis of metastatic or recurrent gastric adenocarcinoma that was previously treated with, though not necessarily resistant to, cytotoxic chemotherapy. The patients were required to have a Eastern Cooperative Oncology Group (ECOG) performance status ≤2, normal blood counts, normal renal and hepatic functions, no history of anaphylaxis and no peripheral neuropathy of any origin. Patients had to have received at least one chemotherapy regimen, either as adjuvant treatment or for metastatic disease. Patients could have received prior 5-FU, provided it was administered in intravenous bolus or in oral form, but not with paclitaxel or docetaxel. Patients had to have fully recovered from the toxic effects of previous chemotherapy except for alopecia.

### Treatment plan

The treatment consisted of docetaxel 75 mg/m^2 ^on day 1 in a 1-h infusion followed by 5-FU in continuous infusion from day 1 to day 5, according to the escalating dose levels. The starting dose of 5-FU was 250 mg/m^2^/day for 5 days. In the absence of DLT, dose escalation in additional cohorts continued at a dose increase of 250 mg/m^2^/dose. Traditional phase I methodology was employed in assessing dose-limiting toxicity (DLT) and MTD. Patients were to be treated at the same dose level in groups of three. If no DLT, defined as febrile neutropenia and/or grade 3/4 toxicity of any other kind apart from alopecia, occurred, the next 3 patients were treated at the next higher dose level. If one DLT occurred, 3 additional patients had to be treated at the same dose level. If 2 or more DLTs occurred at a given dose level, the MTD would be considered to be reached and the dose escalation had to be stopped. The dose just below would be considered to be the recommended dose for further evaluation in phase II trials.

Only the first cycle of treatment was evaluated to determine DLT. However, to adequately determine to safety of this combination, the recruitment of further patients at the recommended dose level was planned in a subsequent phase II study [16]. All patients received a standard supportive regimen consisting of dexamethasone and 5-HT3 inhibitors. The use of hematological growth factors was not allowed during the first cycle of treatment, but was permitted thereafter among patients who had one episode of febrile neutropenia or infection according to the guideline provided by American Society of Clinical Oncology [17].

### Patient evaluation

Toxicity was graded according to National Cancer Institute Common Toxicity Criteria for adverse events (NCI-CTC) [18]. Dose modifications were planned for toxicity. The primary objective of the study was to define the MTD of the regimen under investigation. Evaluation at baseline and during the study included a medical history, data on toxicity and physical examination every courses.

## Results

### Patient characteristics

Sixteen patients received a total of 45 cycles of treatment (median 3, range 1–6). Patient characteristics are listed in Table 1. All patients previously received one cytotoxic chemotherapy regimen, with 88% having received prior fluoropyrimidines. Previous chemotherapy was commonly administered as adjuvant treatment (4 patients, 25%) or for metastatic disease (12 patients; 75%). Twelve patients (75%) had no measurable lesions to evaluate treatment efficacy.

### Toxicity

All patients were evaluable for toxicity. Of three patients enrolled on dose level 1 (5-FU 250 mg/m^2^/day for 5 days), none experienced DLT. On dose level 2 (5-FU 500 mg/m^2^/day for 5 days), a 63-year-old male with peritoneal dissemination developed grade 3 stomatitis and fatigue. Three additional patients were enrolled at this dose level did not experience DLT. Dose escalation thus proceeded to dose level 3 (5-FU 750 mg/m^2^/day for 5 days). One of the 3 initial patients at this level, a 57-year-old male with peritoneal dissemination developed grade 3 stomatitis and another patient, a 60-year-old male with liver metastasis had febrile neutropenia. Because two out of 3 patients had DLTs, the MTD was reached at dose level 3. Dose level 2 was therefore the recommended regimen with docetaxel 75 mg/m^2 ^on day 1 and 5-FU 750 mg/m^2^/day in a 5-day continuous infusion.

Toxicity analysis is based on 16 patients and 45 cycles of treatment. Table 2 summarizes DLTs per patients and per cycles. No treatment-related death was observed.

## Discussion

This phase I trial determined the recommended phase II dose of 5-FU to be 500 mg/m^2^/day as a 5-day continuous infusion when combined with 75 mg/m^2 ^docetaxel every 3 weeks. DLTs included stomatitis, neutropenia, diarrhea, and asthenia. Toxicity in our study did not differ significantly from that seen in other phase I studies that evaluated docetaxel combined with 5-FU continuous infusion. A phase I trial showed that docetaxel at 85 mg/m^2 ^with 5-FU given in continuous infusion over 5 days at 750 mg/m^2^/day was tolerable without any major mucosal toxicity or any substantial increase in docetaxel-induced myelotoxicity [12]. Ando *et al*. [13] determined the MTDs of docetaxel and 5-FU on this schedule to be 50 mg/m^2 ^and 500 mg/m^2^/day, respectively, and neutropenia to be the primary DLT, with diarrhea also dose limiting. Using a similar schedule, Van den Neste *et al*. [14] reported the MTDs of docetaxel and 5-FU to be 85 mg/m^2 ^and 750 mg/m^2^/day, with diarrhea, stomatitis and neutropenia as DLTs.

Because of the relatively high incidence of hematologic toxicity induced by docetaxel, it was felt that only a moderately myelotoxic agent could be added as a combination. 5-FU given in continuous infusion could be an interesting option since it is known to induce very little myelotoxicity, if any.

With the addition of 5-FU, the main changes in the non-hematologic toxicity were observed in the occurrence of stomatitis and diarrhea, and in the appearance of hand-foot syndrome. All three toxicities are known to be associated with 5-FU. Hawkins *et al*. [15] compared docetaxel with the addition of irinotecan or 5-FU in a randomized phase II study. Both docetaxel/irinotecan and docetaxel/5-FU were well-tolerated with acceptable toxicity profiles. Three-drug combination of docetaxel, 5-FU and cisplatin for patients with gastric cancer was also investigated by some authors. Roth *et al*. [19] administered combination chemotherapy with docetaxel 85 mg/m^2^, cisplatin 75 mg/m^2^, and 5-FU 300 mg/m^2^/day on days 1–14, resulting in a 51% objective response. The major toxicity was neutropenia which reached grade 3 or 4 in 79% of 52 patients. Ajani *et al*. [20] evaluated a combination of docetaxel, cisplatin, and 5-FU (DCF) for chemotherapy-naïve patients with advanced gastric cancer. They achieved a response rate of 39% and median survival of 10.2 months, and toxicity was manageable. It is apparent that the more complex a chemotherapy regimen, the more toxic and difficult for patients to tolerate. Although cisplatin is often used in combination with other agents, it is well known that cisplatin is associated with significant toxicity and usually requires high level of clinical monitoring and supportive care including intensive intravenous hydration. Thuss-Patience *et al*. [21] recently reported that docetaxel/5-FU had similar efficacy to epirubicin/cisplatin/5-FU (ECF) and even to DCF.

Since nearly 80% of the patients did not have measurable disease, an efficacy assessment could not be performed in this patient population. Our randomized phase II trial with docetaxel/5-FU versus paclitaxel/5-FU is currently ongoing [16].

## Conclusion

In conclusion, the recommended dose of this combination is 75 mg/m^2 ^docetaxel on day 1 immediately followed by a 5-day continuous infusion of 5-FU 500 mg/m^2^/day. Further evaluation of efficacy and safety is currently underway in a randomized phase II trial of 5-FU combined with docetaxel versus paclitaxel.

## Competing interests

The author(s) declare that they have no competing interests.

## Authors' contributions

SHP collected the data, performed the statistical analysis and drafted the manuscript. SB, EKC, JHL, WKL and MC followed the patients. DBS designed the study and helped with the manuscript. All authors read and approved the final manuscript.

## Pre-publication history

The pre-publication history for this paper can be accessed here:



## Figures and Tables

**Table 1:** Patient characteristics

Number of patients	N = 16	
Age		
Median	59	
Range	36–73	
Male:female	14:2	
ECOG performance status		
0	6	37%
1	8	50%
2	2	13%
Disease status		
Primary metastatic	12	75%
Locally-advanced	4	25%
Prior chemotherapy		
Uracil-tegafur	4	25%
5-FU bolus + folinic acid	1	6%
5-FU + cisplatin	4	25%
Epirubicin + cisplatin + capecitabine	5	31%
Epirubicin + doxorubicin + cisplatin	2	13%

**Table 2:** Dose-limiting toxicities (DLTs) according to dose level.

Dose level (5-FU/day)	Pts/cycles	After first cycle	After all courses (pts/cycles)				
			
			Neutropenia	FN^1^	stomatitis	diarrhea	asthenia
Level 1 (250 mg)	3/4	None			1/1		1/1
Level 2 (500 mg)	10/35	Stomatitis (1)	4/5		6/11	1/1	4/7
Level 3 (750 mg)	3/4	Stomatitis (1) FN (1)	2/2	1/1	2/2	1/1	1/1

1 FN denotes febrile neutropenia.
